# Penetrance and expressivity of the R858H *CACNA1C* variant in a five‐generation pedigree segregating an arrhythmogenic channelopathy

**DOI:** 10.1002/mgg3.476

**Published:** 2018-10-21

**Authors:** R. J. McKinlay Gardner, Ian G. Crozier, Alex L. Binfield, Donald R. Love, Klaus Lehnert, Kate Gibson, Caroline J. Lintott, Russell G. Snell, Jessie C. Jacobsen, Peter P. Jones, Kathryn E. Waddell‐Smith, Martin A. Kennedy, Jonathan R. Skinner

**Affiliations:** ^1^ Cardiac Inherited Disease Group Auckland New Zealand; ^2^ Genetic Health Service New Zealand (South Island Hub) Christchurch Hospital Christchurch New Zealand; ^3^ Clinical Genetics Group, Department of Women's and Children's Health University of Otago Dunedin New Zealand; ^4^ Department of Cardiology Christchurch Hospital Christchurch New Zealand; ^5^ Department of Paediatrics Christchurch Hospital Christchurch New Zealand; ^6^ Department of Paediatrics University of Otago Christchurch New Zealand; ^7^ LabPlus Auckland City Hospital Auckland New Zealand; ^8^ School of Biological Sciences University of Auckland Auckland New Zealand; ^9^ Department of Physiology and HeartOtago University of Otago Dunedin New Zealand; ^10^ Department of Cardiology Auckland City Hospital Auckland New Zealand; ^11^ Department of Pathology and Biomedical Science University of Otago Christchurch New Zealand; ^12^ Present address: Department of Pathology Sidra Medicine Doha Qatar

**Keywords:** arrhythmia, CACNA1C, expressivity, long QT, penetrance

## Abstract

**Background:**

Isolated cardiac arrhythmia due to a variant in *CACNA1C *is of recent knowledge. Most reports have been of singleton cases or of quite small families, and estimates of penetrance and expressivity have been difficult to obtain. We here describe a large pedigree, from which such estimates have been calculated.

**Methods:**

We studied a five‐generation family, in which a *CACNA1C *variant c.2573G>A p.Arg858His co‐segregates with syncope and cardiac arrest, documenting electrocardiographic data and cardiac symptomatology. The reported patients/families from the literature with *CACNA1C *gene variants were reviewed, and genotype–phenotype correlations are drawn.

**Results:**

The range of phenotype in the studied family is wide, from no apparent effect, through an asymptomatic QT interval prolongation on electrocardiography, to episodes of presyncope and syncope, ventricular fibrillation, and sudden death. QT prolongation showed inconsistent correlation with functional cardiology. Based upon analysis of 28 heterozygous family members, estimates of penetrance and expressivity are derived.

**Conclusions:**

These estimates of penetrance and expressivity, for this specific variant, may be useful in clinical practice. Review of the literature indicates that individual *CACNA1C *variants have their own particular genotype–phenotype correlations. We suggest that, at least in respect of the particular variant reported here, “arrhythmogenic channelopathy” may be a more fitting nomenclature than long QT syndrome.

## 1 INTRODUCTION

Inherited cardiac rhythm dysfunction is typically the consequence of an ion channelopathy expressed in the cardiomyocyte (Betzenhauser, Pitt, & Antzelevitch, 2015). The most commonly recognized group of disorders are the long QT syndromes (LQTS), in their several genetic forms due to mutation in one of these ion channel genes: *KCNQ1*, *KCNH2*, *KCNE1*, *KCNE2*, *KCNJ2*, *KCNJ5*, *SCN5A*, *SCN4B*, *ANK2*, *CAV3*, *AKAP9*, *SNTA1*, *CALM1*, *CALM2*, and *CACNA1C*. We describe a family presenting with syncope, dysrhythmia including ventricular fibrillation, and sudden cardiac death, segregating with an autosomal dominant R858H variant in the *CACN1AC* gene. While some family members had borderline or moderate QT prolongation, this bore no clear relationship to symptomatology. The five‐generation pedigree is of sufficient size that useful broad estimates of penetrance and expressivity can be derived.

## 2 MATERIALS AND METHODS

### 2.1 Ethical compliance

The family members in this report are enrolled in the New Zealand Cardiac Inherited Disease Registry; at enrollment, they give their permission for de‐identified publication related to their condition.

### 2.2 Family report

**Figure 1:**
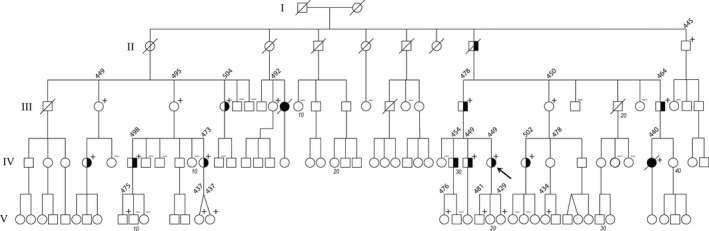
Pedigree of the family. Arrow = index patient. + = proven heterozygous for the p.R858H mutation; ‒ = homozygous normal at codon 858. Individuals IV:30 and IV:34 are obligate heterozygotes by pedigree. Half‐filled symbol = symptomatic, typically syncopes; filled symbol = sudden death. Longest recorded QT interval at routine electrocardiography, when otherwise in good health, indicated as diagonal number above symbol (see Table 1 for ranges). No annotation = not known on family report to have presented with relevant symptomatology, but have not been studied by us. The descendants of those identified not to carry the *CACNA1C* mutation are not shown. Marker numbers below symbol show every tenth person per generation

The pedigree is shown in Figure 1. The family is of European (Anglo‐Celtic) ancestry, and of New Zealand residence. The index patient (IV:32) presented at age 10, having suffered an out‐of‐hospital cardiac arrest; she was asleep in her bed at the time, her parents heard what they called a “death rattle,” and they resuscitated her. Upon the arrival of an ambulance, ventricular fibrillation was recorded, and defibrillation carried out. She was started on beta‐blocker therapy. She had a second cardiac arrest at age 11, again at night, her father performing cardiopulmonary resuscitation until an ambulance with a defibrillator arrived. Following this episode, an implantable cardioverter‐defibrillator (ICD) was inserted. An arrest at age 21 triggered defibrillation. At 23, having failed to take her beta‐blocker the night before, she went into ventricular fibrillation and was only reverted after the fourth shock, of 41 joules. Now in her thirties, she has been stable for some years. QT interval prolongation has never been observed on the many surface EKG studies she has had, including 24‐hr Holter and exercise EKGs; only in the shock electrogram immediately prior to an event was the QT prolonged.

The knowledge that she had had, at the time, a cousin dying suddenly at young age (36), and subsequently another cousin dying at age 26, led to other family members being assessed and, in due course, a *CACNA1C *variant to be discovered, as detailed below. A total of 40 individuals underwent evaluation by a cardiologist, and genetic testing. A total of 26 were variant‐positive; none of those who were variant‐negative had a history of syncope or a prolonged QT interval. The clinical findings in those shown to be heterozygous for the variant are recorded in Table 1. To the best of (incomplete) family knowledge, none of individuals I:1–2 and II:1–6, born in the earlier twentieth century, had presented with syncopal episodes or other possible manifestation of a dysrhythmia, and I:1 and I:2 had lived into their 70s or 80s. Individual II:7 had had two witnessed collapses: once following a running race, and once as he chased his young son (III:17) around the front yard.

**Table 1:** Pedigree data, with respect to 26 known/presumed^a^ heterozygotes

Individual	Age^b^	QTc range^c^	Symptomatology^d^
II:7	d. 74	–	Syncopes ×2 by distant anecdote
II:8	75	445	Asymptomatic
III:2	66	449–738	Asymptomatic
III:3	64	472–495	Asymptomatic
III:4	61	442–504	Syncope ×1, multiple pre‐syncopes
III:8	70	492	Asymptomatic
III:9	36	–	None known prior to sudden death
III:17	61	472–478	Several syncopes, none since age ~50
III:18	65	450–584	Asymptomatic
III:22	71	418–464	multiple syncopes since young adulthood
IV:4	41	e	“Multiple faints in adolescence”
IV:6	41	440–498	Syncope ×2 associated with palpitations
IV:11	29	473	Syncopes ×2 age 10 and 13
IV:30	34	436–454	Single syncope with convulsions
IV:31	32	429–449	“Blackouts as a boy”; adult syncope ×1
IV:32	30	410–449	Index case
IV:33	43	486–502	Asymptomatic
IV:34	46	476–478	Syncope ×1 as adult
IV:39	d. 26	440 450–510^f^	Syncopes, sudden death
V:9	9	475	Asymptomatic (young child)
V:14^g^	1	368–437	Asymptomatic (infant)
V:15 g	1	422–437	Asymptomatic (infant)
V:16	8	441–476	Asymptomatic (young child)
V:19	6	422–481	Tetralogy of Fallot (young child)
V:21	2	379–429	Asymptomatic (infant)
V:25	24	433–434	Asymptomatic

a Presumed on the basis of pedigree position, as obligate heterozygotes; or, having suffered sudden death.

b At time of study or death.

c In ms, from automated reading of routine records at rest, the observations often covering several years. In a few, only one record available. The only two substantially raised QTc intervals (in III:2 and III:18) were in the context of concomitant heart disease otherwise (see text).

d Relevant to a possible dysrhythmia.

e Reported to have been elongated during third pregnancy; normal interval on stress test post partum. Actual EKG data not available.

f Holter study (see Supporting information Appendix S1). Otherwise, routine EKGs had been normal.

g Diamniotic dichorionic female twins; zygosity uncertain.

III:9 This previously fit and healthy woman, a first cousin once removed of the index patient, died suddenly after jumping into a swimming pool, at age 36. The brief autopsy report noted the heart muscle to show a “diffuse mottling in color,” and on histology, there was “an increase in fibrosis with edema, together with a diffuse infiltrate by inflammatory cells, mainly lymphocytes.” This was interpreted as “acute heart failure associated with an idiopathic cardiomyopathy.” No archived tissue was available for retrospective review. Given a normal heart weight (350 g, 5 ft 9 in female), and absent any reference to chamber dilation or myocardial hypertrophy, we have reservations about this interpretation.

IV:39 This young woman, a first cousin of the index patient, had had collapses of brief duration from teenage, most notably on the performance field as a marching girl. The single reference we have to a routine EKG report noted a QTc interval of 440 ms; a QTc ranging from 450 to 510 ms had been demonstrated on Holter study (full report in Supporting information Appendix S1). She had begun beta‐blocker therapy, but ceased taking the medication for the course of her first pregnancy, and had planned to recommence at 12 months after the birth. At 11 months after the birth, at age 26 years, she had apparently inadvertently slept in, and received a phone call to remind her of the time, but did not answer. She was later found dead with the phone in her hand. On the assumption of a known cause, no autopsy was done.

### 2.3 Electrophysiology

**Figure 2:**
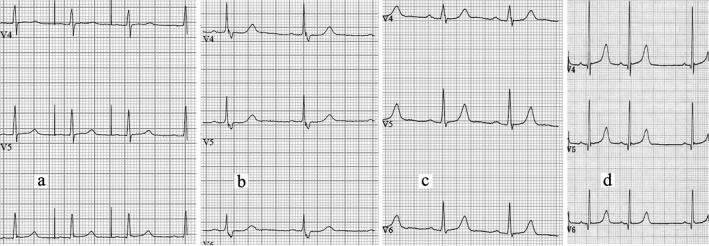
Representative EKGs (V4‐V6 precordial leads) of (a) the index patient (IV:32), (b) her father (III:17), (c) her great‐uncle (II:8), and (d) 9‐year‐old distant cousin (V:9). Corrected QT intervals (automated calculation) in the first three traces are, respectively, 449 ms (note artefact due to atrial pacemaker), 472 ms, and 445 ms; in V:9, the QTc measured with digital callipers is 475 ms. (The upper limit of normal for a female is 460 ms, and for a male, 440 ms.) Similar EKGs on all studied family members, and replicates of these four, are provided in Supporting information Appendix S1

All proven heterozygotes have had resting 12‐lead EKGs, often on many occasions, and over several years. The EKGs were reviewed by I.G.C. and J.R.S. For consistency in this study, we have reported the automated QTc measurements, and have taken care to ensure that these were representative, since such automated measurements can sometimes be erroneous with unusual T wave morphology. Representative precordial (chest leads V4‐V6) traces are shown in Figure 2, and similar EKG images on all available studied patients are provided in the Supporting information Appendix S1. Selected patients (III:17, IV:11, IV:31, and IV:32) had an exercise EKG; Holter 24‐hr recordings were performed in IV:32 and IV:39. The rhythm strips from two events in the index patient in which her ICD was triggered (shock electrograms) are shown in the Supporting information Appendix S1.

### 2.4 Molecular genetics

Genomic DNA was prepared from the peripheral blood of family members III:17, IV:31, and IV:32. Initial genetic testing for long LQT types 1 (*KCNQ1*), 2 (*KCNH2*), 3 (*SCN5A*), 5 (*KCNE1*), and 6 (*KCNE2*) had been uninformative. Whole exome sequencing was then undertaken, on the Illumina XTen system (Garvan Institute, Sydney, Australia), generating 909–929 million 150‐nucleotide‐long paired‐end reads for each sample. Reads were aligned to the GRCh37.p13 human genome assembly using Burrows‐Wheeler aligner, version 0.7.12 (Li & Durbin, 2009), to obtain average autosomal coverages between 33.5 and 34.5. Single‐nucleotide and indel variants were identified using the GATK tools HaplotypeCaller and GenotypeGVCFs according to the GATK best practices workflow, version 3.4‐0 (DePristo et al., 2011). Variants were annotated with controlled‐vocabulary terms describing the variant's consequence, as well as observation frequencies in global populations and our in‐house cohort of individuals without cardiac conditions, using custom scripts. Only high‐confidence variants observed at global or local minor allele frequencies below 0.05, affecting protein primary structure, and concordantly observed with autosomal dominant inheritance pattern in all three family members, were further considered. A total of 177 variants met these criteria; of these, four variants located in three of 133 candidate genes were prioritised for interpretation based on physiological function, tissue expression, and variant impact severity.

The exome sequence of family member III:18 was determined as part of an independent study (Lacey et al., 2017), her membership of the present family at the time unknown. A total of 24 million 102 nucleotides‐long paired‐end reads were generated using the Illumina HiSeq system (Macrogen, Seoul, Republic of Korea). Read alignment and variant identification was performed as described above.

Having identified a variant (see below), family members presumed on clinical grounds to be affected, and those at risk by virtue of their position on the pedigree, were tested for the variant by PCR amplification of the genetic interval harboring the variant using DNA prepared from peripheral blood samples, followed by Sanger sequencing. Of the two cases of sudden cardiac death, no pathology tissue was available for a retrospective study in the case of III:9; the Guthrie card of patient IV:39 was retrieved from 1973 storage, and DNA isolated using the QIAamp DNA mini kit (Qiagen, Germany).

### 2.5 Pedigree analysis

Many family members were reviewed on the occasion of a dedicated 2‐day genetic clinic in 2016, and available medical records were studied, and the pedigree structure confirmed. The individuals displaying a phenotype and, if so, of what manifestation, were noted (Table 1). Proof of genotype is lacking in III:9, who had suffered sudden death, but we have made the reasonable assumption of likely heterozygosity, given an a priori 0.5 risk on pedigree and the a posteriori observation, the ambiguous autopsy report of “idiopathic cardiomyopathy” notwithstanding. Segregation analysis was conducted on elementary principles.

## 3 RESULTS

**Figure 3:**
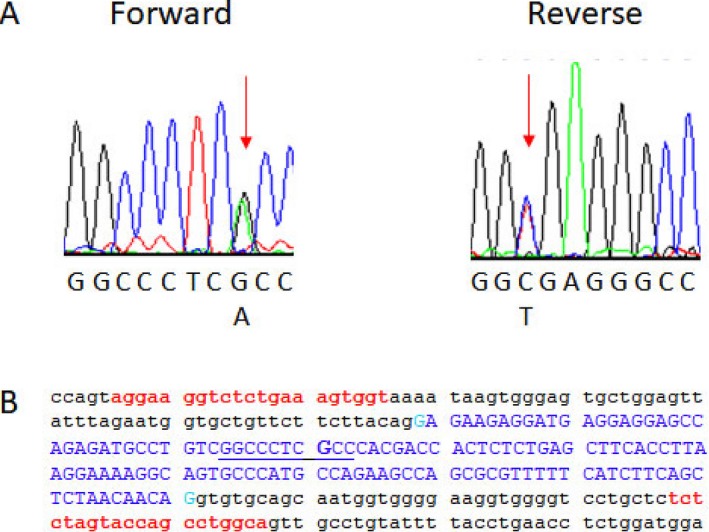
Electropherograms and sequence of the c.2573G>A mutation. Panel A shows the Sanger‐based electropherograms for the forward and reverse directions of a representative carrier of the NM_000719.6:c.2573G>A mutation in the *CACNA1C *gene. The vertical red arrows show the location of the heterozygous mutation event; the base sequences are shown beneath the electropherograms. Panel B shows the DNA sequence of exon 19 of the *CACNA1C* gene. The lower case letters indicate intronic sequence; the dark blue upper case letters represent exonic sequence; the light blue letters represent the proximal and distal bases of the exon; the forward and reverse primers for PCR amplification of this exon are shown in red; the bases in the electropherograms of panel A are underlined in blue; and the nucleotide in question, c.2573G, is shown in bold blue

A G‐to‐A transition in exon 19 of the *CACNA1C* gene (NM_000719.6:c.2573G>A; NP_000710.5: p.Arg858His; rs786205753; OMIM accession 114,205) was identified in family members III:17, IV:31, and IV:32; a representative electropherogram of a heterozygous family member is shown in Figure 3. This variant is absent in the genome aggregation database (gnomAD; https://exac.broadinstitute.org) cohorts, corresponding to an allele frequency below 3.6 × 10^‒6^ (Lek et al., 2016); it is listed in Ensembl (rs786205753), but with no frequency noted, presumably attesting to its rarity. A single individual carrying the same variant was coincidentally identified in the New Zealand genome sequencing collaboration cohort, and she was subsequently identified as family member III:18. The variant substitutes an arginine residue, which is fully conserved in all vertebrates sequenced to date, for a histidine residue (Casper et al., 2018). Pathogenicity is interpreted as “deleterious” on SIFT, and as “probably damaging” on PolyPhen. Inheritance is autosomal dominant.

Every person who self‐reported relevant symptomatology (typically syncopal episodes) proved to be heterozygous; some heterozygotes denied ever having had symptoms (Table 1). One of the two suffering sudden death (IV:39) proved also to have been heterozygous on retrospective Guthrie card analysis. A LOD score confined to analysis of clinically affected known heterozygotes reaches 3.61 at  θ= 0. The QTc intervals, where known, are listed in Table 1; these range from normal, through slightly long, to more substantially long. None showed Brugada syndrome characteristics. Four symptomatic family members, including the index patient, underwent exercise testing. All had normal QT intervals at 100 bpm during the recovery phase: III:17, 300 ms; IV:11, 340 ms; IV:31, 370 ms; and IV:32, 320 ms (normal range up to 380 ms; Swan et al., 1999). The penetrance of the variant, at the level of overt symptomatology, was 46% (13/28). Of those 13 presumed or proven heterozygotes manifesting signs, in 77% (10/13) this was limited to one or more syncopal episodes, and 23% (3/13) suffered either sudden cardiac death or, in the index patient, aborted cardiac death (Table 2). No extra‐cardiac manifestations co‐segregated with the variant.

**Table 2:** Estimates of penetrance and expressivity^a^

Total heterozygotes assessed	28
Asymptomatic	15 (54%)
Syncopal episode(s)	10 (36%)
Major dysrhythmia^b^	1 (4%)
Sudden death	2 (7%)
Any symptomatic manifestation	13 (46%)
Any symptomatic manifestation (proband excluded)	12 (43%)
Any symptomatic manifestation (infants/young children and proband excluded)	12 (55%)
QTc>500 ms, female, or>480 ms, male^c^	5 (22% of 23 with EKG data)

a See also Discussion for different viewpoints of assessments of the data.

b Ventricular fibrillation; presumed aborted sudden death (proband).

c In the context of normal health otherwise, on routine resting EKG; highest recorded figure.

## 4 DISCUSSION

The *CACNA1C *gene can be expressed in a number of isoforms; here, we consider its expression in the cardiomyocyte. The first report of its role in the control of heart rhythm appeared in 2004, when Splawski et al. described what came to be known as Timothy syndrome, in which a LQTS coexists with a multi‐system manifestation, including intellectual deficiency, syndactyly, immunodeficiency, and a distinctive facies. Timothy syndrome proved almost always to be on the basis of a recurrent mutation p.G406R (within the alternatively spliced exon 8A), with one or two examples of G402R, G402S, I1166T, and A1473G substitutions also known. These cases typically represented a de novo dominant mutation in the family, and with death occurring in infancy in many (Tester & Ackerman 2014; Boczek et al., 2013; Gillis et al., 2012; Walsh et al., 2018). The only familial examples were siblings with p.G402S born to a mosaic, unaffected father, and siblings with p.G406R born to a mosaic, unaffected mother (Fröhler et al., 2014; Splawski et al., 2004).

**Figure 4:**
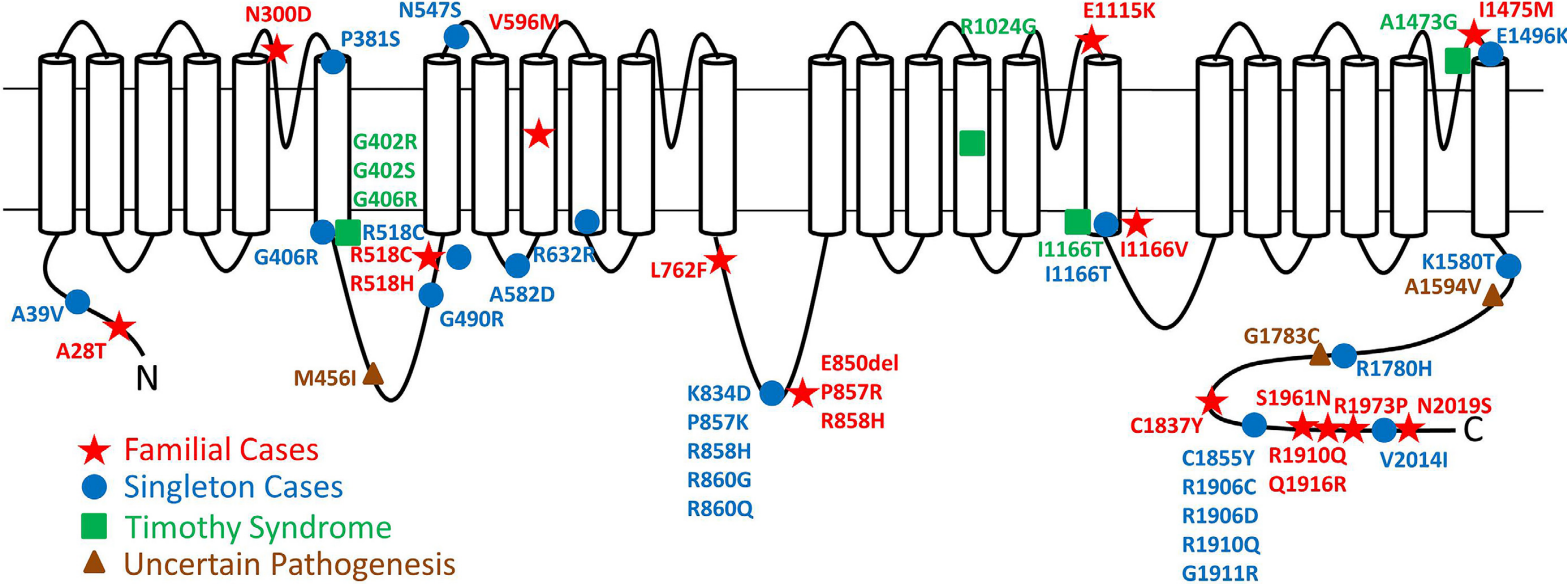
Diagram of the CACNA1C protein transmembrane domains and loops, with mutation sites of the cases listed in Tables 3, 4, 5, 6, 7 indicated. The mutation in the present family, R858H, resides within the loop between transmembrane domains II and III

The first recognition of a *CACNA1C*‐associated isolated dysrhythmia came from Antzelevitch et al. (2007), who identified two such patients in a screen of 82 individuals diagnosed with Brugada syndrome. The first report of a *CACNA1C* variant in isolated LQTS is due to Boczek et al. (2013), at which time the previously applied nomenclature LQTS type 8 was confirmed; and several further patients/families with LQT or other dysrhythmia have since been reported in the 2010s, as listed in Tables 3, 4, 5, 6, 7. We have separated these into those in which an observation of co‐segregation was ipso facto supportive of true pathogenicity, versus single observations in some of which a definitive conclusion might not necessarily be drawn, albeit that in several, sophisticated cell biology studies were surely compelling. Some *CACNA1C* variants were associated with a Brugada EKG picture, as mentioned above; in a few, short QT and early repolarisation syndrome was recognized. A long QT interval was the observation in a majority. The sites of variation are illustrated in Figure 4, distinguishing those located within in loops (loops comprise approximately three‐quarters of the CACNA1C protein), and those actually within transmembrane domains (comprising one‐quarter of the protein). Almost all variants reside within a loop, possibly reflecting a greater sensitivity to functional compromise of a transmembrane domain.

**Table 3:** Recorded *CACNA1C *mutations and associated phenotypes: familial cases with co‐segregation

Mutation	Reference	Phenotype
A28T	Wemhöner et al. (2015)	LQT
N300D^a^	Béziau et al. (2014)	SQT, Brugada
R518C	Boczek, Ye, et al. (2015)	LQT, HCM, CHD
“	Boczek, Ye, et al. (2015)	LQT, HCM
R518H	Boczek, Ye, et al. (2015)	LQT, HCM
V596M^b^	Zhu et al. (2018)	SSS
L762F	Landstrom et al. (2016)	LQTS
P857R	Boczek et al. (2013)	LQT
R858H	Fukuyama et al. (2013)	Dysrhythmia, fam hx SCD
“	Present family	Dysrhythmia, fam hx SCD, CHD
E850del	Burashnikov et al. (2010)	ERS
“	Sutphin et al. (2016)	SUDY
E1115K	Burashnikov et al. (2010)	Brugada
I1166V	Wemhöner et al. (2015)	LQT
I1475M	Wemhöner et al. (2015)	LQT
C1837Y	Burashnikov et al. (2010)	Brugada, SQT
R1910Q	Fukuyama et al. (2013)	Brugada
Q1916R^c^	Liu et al. (2017)	ERS, SCD
S1961N^d^	Nieto‐Marín et al. (2018)	LQT
R1973P^e^	Chen et al. (2017)	Short QT, ERS
N2019S	Sutphin et al. (2016)	SUDY

Notes

CHD: congenital heart defect; ERS: early repolarization syndrome; fam hx: family history; HCM: hypertrophic cardiomyopathy; LQT: long QT; SCD: sudden cardiac death; SQT: short QT; SSS: sick sinus syndrome; SUDY: sudden unexplained death of the young.

a Some family members also carried *SCN5A *mutation Q1695*, complicating interpretation.

b Some family members also carried *TTN* R16472H, complicating interpretation.

c Some family members also carried *SCN5A *mutation R1193Q, complicating interpretation.

d Some family members also carried *SCN5A *mutation R1644H, complicating interpretation.

e Father and daughter also carried *DES* variant E234K and *MYPN* variant R989H, complicating interpretation.

**Table 4:** Recorded *CACNA1C *mutations and associated phenotypes: singleton cases with indicative family history

Mutation	Reference	Phenotype
A39V	Antzelevitch et al. (2007)	Brugada, SQT; fam hx SCD
G490R	Antzelevitch et al. (2007)	Brugada, SQT; fam hx SCD
“	Burashnikov et al. (2010)	Brugada, SQT
R518C	Seo et al. (2018)	CHD, LQT; fam hx SCD
R858H	Fukuyama et al. (2013), Fukuyama, Wang, et al. (2014)	Asymptomatic; fam hx SCD
K1580T	Kojima et al. (2017)	CHD, VF, TdeP; fam hx LQT

Note

CHD: congenital heart defect; fam hx: family history; LQT: long QT; SCD: sudden cardiac death; SQT: short QT; TdeP: torsades de pointes; VF: ventricular fibrillation.

**Table 5:** Recorded *CACNA1C *mutations and associated phenotypes: Singleton Cases (or Familial Status not Indicated)

Mutation	Reference	Phenotype
P381S	Fukuyama, Wang, et al. (2014)	LQT
G406R	Sepp et al. (2017)	LQT, not TS
“	Hiippala, Tallila, Myllykangas, Koskenvuo, and Alastalo (2015)	LQT, not TS
N547S	Fukuyama et al. (2013)	Brugada
A582D	Fukuyama, Wang, et al. (2014)	LQT
R632R	Fukuyama et al. (2013)	VF
T171M^a^	Narula, Tester, Paulmichl, Maleszewski, and Ackerman (2015)	SCD
K834D	Boczek et al. (2013)	syncope, LQT
P857K	Boczek et al. (2013)	LQT
R858H	Fukuyama et al. (2013), Fukuyama, Wang, et al. (2014)	Syncope, bradycardia, LQT
R860G	Wemhöner et al. (2015)	LQT
R860Q	Seo et al. (2018)	LQT
I1166T	Wemhöner et al. (2015)	LQT
E1496K	Wemhöner et al. (2015)	LQT
R1780H	Fukuyama et al. (2013)	Brugada
C1855Y	Fukuyama et al. (2013)	Brugada
R1906C	Boczek et al. (2013)	LQT
R1906D	Boczek et al. (2013)	palpitations, syncope, LQT
R1910Q	Fukuyama et al. (2013)	Brugada
G1911R	Hennessey et al. (2014)	LQT, VT, microcephaly, seizures, spastic diplegia
V2014I	Burashnikov et al. (2010)	Brugada

Notes

LQT: long QT; SCD: sudden cardiac death; TS: Timothy syndrome; VF: ventricular fibrillation.

a Also carried *MYH7 *variant A1744S, complicating interpretation.

**Table 6:** Recorded *CACNA1C *mutations and associated phenotypes: Timothy Syndrome (TS)

Mutation	Reference	Phenotype
G402S	Splawski et al. (2005)	TS
“	Fröhler et al. (2014)	TS^a^
G406R	Splawski et al. (2004)	TS^b^
“	Walsh et al. (2018)	TS^c^
“	Diep and Seaver (2015)	Partial TS
“	Landstrom et al. (2016)	TS
G406R (mos)	Baurand et al. (2017)	Long QT, partial TS
S643F	Ozawa et al. (2018)	Long QT, incomplete TS
R1024G	Kosaki, Ono, Terashima, and Kosaki (2018)	Incomplete TS, QT normal
I1166T	Boczek, Miller, et al. (2015)	TS
“	Wemhöner et al. (2015)	TS
A1473G	Gillis et al. (2012)	TS

a Two affected siblings of an unaffected mosaic father.

b Series of 13 TS cases, including two affected siblings of an unaffected mosaic mother.

c Series of 5 TS cases.

**Table 7:** Recorded *CACNA1C *mutations and associated phenotypes: Uncertain Pathogenicity

Mutation	Reference	Phenotype
M456I	Fukuyama, Wang, et al. (2014)	Long QT
A1594V^a^	Wang et al. (2017)	Inverted T waves
G1783C	Fukuyama, Ohno, et al. (2014)) and Fukuyama, Wang, et al. (2014)	Long QT

a Some family members also carried MYH7 variant V878A, complicating interpretation.

One other multi‐generational family is known with precisely the same variant, c.2573G>A, R858H, as in the present case: Fukuyama et al. (2013), Fukuyama, Wang, et al. (2014) describe a 4‐member 3‐generation Japanese family, in which the 54‐year‐old proposita presented with nocturnal “electrical storm.” (Fukuyama et al. refer also to c.2339G>A, likely referring to a different transcript isoform, but it is clear from their illustrated electropherogram that both variants affect the same genome coordinate). Her father had died in his sleep at age 47, a presumed sudden cardiac death, and her two young adult daughters were asymptomatic, with QTc intervals of 480 ms (slightly prolonged) and 444 ms (normal), respectively. Two other R858H singleton patients (one symptomatic; the other an asymptomatic child found on routine screening, but whose uncle had suffered sudden cardiac death) are also recorded in Fukuyama et al. (2013), Fukuyama, Wang, et al. (2014).

There is considerable evidence supporting the pathogenicity of this R858H variant, given the following: its identification in the two LQT8 pedigrees, one Japanese and one New Zealand, and at least one further Japanese symptomatic singleton patient, and with co‐segregation with phenotypic symptomatic status in the New Zealand family; from observations of in vitro biophysical assays in which the variant had been expressed in Chinese hamster ovary cells (Fukuyama, Ohno, et al., 2014); from computer simulations of the dynamics of human ventricular cells (Bai et al., 2017; Fukuyama, Ohno, et al., 2014); and from the population analyses conducted by ourselves as outlined above.

Further support for the pathogenicity of the R858H substitution lies in its relative location within the CACNA1C (calcium voltage‐gated channel subunit alpha1 C′) protein, within the cytosolic loop between transmembrane domains II and III (Figure 4). This II–III loop contains a PEST sequence/domain, important for the turnover of the channel. Previous studies of R858H, and of the neighboring LQTS‐associated mutant p.P857R, describe a gain‐of‐function phenotype plausibly due to a reduction in the internalization and degradation of the channel (Boczek et al., 2013). The increased surface expression results in an increase of the inward calcium current (I_Ca,L_) prolonging the action potential (Fukuyama, Wang, et al., 2014), this implying a functional vulnerability.

A noteworthy feature of the present family is the unremarkable nature of the EKG observations in many, despite the fact of a channelopathy sufficient to predispose to sudden arrhythmic cardiac death. In the index patient, the QTc intervals at rest were repeatedly normal and shortened normally with exercise. In some gene carriers, the QTc was mildly prolonged (Figure 2, Table 1, Supporting information Appendix S1). Two individuals (III:2 and III:18) had more markedly prolonged QTc intervals, but this was in the context of concomitant heart disease otherwise. III:2 had a historic QTc of 449 ms; latterly an interval of 525 ms with associated left bundle branch block; and subsequently 738 ms in the course of an intercurrent acute myocardial infarction. III:18 had a QTc interval of 584 ms in the setting of a stress cardiomyopathy (Chan et al., 2013), but thereafter returning to 450 ms. The QTc interval in the index patient was prolonged only in the setting of the ICD being triggered (shock electrogram, Supporting information Appendix S1). The QTc intervals in the deceased young woman IV:39 had been reported as normal on a stress EKG; on a 24 hr Holter recording (Supporting information Appendix S1), the interval varied from normal to prolonged. We conclude that, in otherwise good health, the QTc interval at routine testing may often be normal or only slightly increased, but that repolarization is vulnerable if other cardiac disease is coincidentally present (one could speculate about asymptomatic heart disease otherwise in patient III:9, who had suffered sudden death, and whose autopsy report is discussed above). One rather obvious practical point emerges: Measurement of the QT interval can be misleading in determining genetic status. The presence of QT prolongation is clearly suspicious, but its absence is not at all reassuring; and unlike most other forms of long QT syndrome, the length of the QT interval appears to bear inconsistent relationship to disease severity, most notably with respect to the two patients with actual (IV:39) or aborted (IV:32) sudden death (Table 1).

The pedigree in the present family is sufficiently large (to our knowledge, the most extensive *CACNA1C* family on record) that useful, albeit broad estimates may be derived for penetrance and expressivity of this specific *CACNA1C* variant. Inevitably much data is anecdotal, and we have accepted such reports as “multiple faints in adolescence,” and “blackouts as a boy,” as likely reflecting at least a presyncope, and probably a syncope. The asymptomatic status of II:1 and II:2 may be less securely held, but they did both live to old ages. An overall penetrance value of 0.46 (13/28) is obtained (Table 2). In the 46% in whom disease is manifest, the expressivity has varied from having had syncopal (or pre‐syncopal) episodes, through severe dysrhythmia, to sudden cardiac death. The estimates of the different levels of expressivity (Table 2) will be useful in managing heterozygous persons. Presumably, these conclusions could be extrapolated to other families in which the same variant has been, or in the future may be, discovered.

We accept that the penetrance values from the segregation analysis might be slight over‐estimates, due to biased ascertainment of the family. A biasing effect in the other direction may be due to the young age of some heterozygotes, who may yet have fully to declare their phenotypic status (penetrance rises to 59%, 13/22, if data from children under 10 years are removed). If the anecdotal historical reports of obligate heterozygotes II:1 and II:2 having been asymptomatic are regarded as too uncertain and are excluded from the calculation, penetrance would increase to 50% (13/26; children included) or 65% (13/20; children excluded). If, as is conventional in segregation analysis, the proband (who suffered aborted sudden death) is excluded, the respective percentages are 46% (12/26) and 60% (12/20). Overall, a fraction in the vicinity of 50%–60% may be valid as a reasonable penetrance estimate. With respect to sudden death, 7% may be an appropriate approximate risk figure, but bearing in mind the proviso due to biased ascertainment of the family, and the fact also of small numbers. Treatment with beta‐blockers has been provided to mutation‐carriers, noting that a meta‐analysis with respect at least to LQTS 1, 2, and 3 showed reductions in risk of variable degree (Ahn et al., 2017), and that the proposita in the Japanese R858H family discussed above was said to respond favorably to beta‐blockade.

What might be the basis of the non‐penetrance in some and varied expressivity in others? Might this simply be a matter of the stochastic disposition of the mutant CACNA1C channel in the sarcolemma? Or, and perhaps more plausibly, might there be an agency of genetic variation at other loci? “Normal variation”—that is, a minor allelic form at another locus or loci insufficient per se to influence channel function—could plausibly comprise a subtle “second hit” effect in the vulnerable setting of a major known pathogenic mutation (Coll et al., 2017; Giudicessi & Ackerman, 2013). Such variation would not be expected to co‐segregate with the major mutation; at least with respect to the 14 channel genes listed above, none are located on the same chromosome (number 12) as is *CACNA1C*. A similar but more palpable basis of reduced penetrance is proposed in Liu et al. (2017), of a family in which some members carried a convincingly pathogenic mutation in another gene (*SCN5A*) which appeared to modulate the effects of a *CACNA1C *p.Q1916R variant, a scenario which these authors refer to as “digenic variation.” Similar pictures are recorded in Nieto‐Marín et al. (2018) and in Zhu, Luo, Jiang, and Liu (2018). Variation elsewhere in the same gene may not, however, necessarily have an effect, as Crotti et al. (2016) show with respect to the *KCNQ1 *(LQTS 1) variant. Other proposed modifying factors, including gender and dietary practice, are reviewed in Giudicessi and Ackerman (2013), Coll et al. (2017), and Liu et al. (2017).

It is of interest that the correlation between *CACNA1C* variant and phenotype is quite tight. The example of Timothy syndrome, with severe extracardiac effects, is notable. Concerning the case of isolated cardiac manifestation, some variants are prone to present a Brugada EKG picture, while others are more in the mold of classic LQT (Tables 3, 4, 5, 7). The variant in our family has, as discussed above, an inconsistent relationship to the QT interval, with only a few having a notably increased length. Some of the males in the present family have had classical exercise‐related syncope (as with LQTS 1), and of the three females who have had cardiac arrest or sudden death, one was typical for LQTS 2, being in the post partum period, and on the phone. These observations lead us to suggest that whilst the family would be conventionally classified amongst the broad spectrum of “long QT syndromes,” in the absence of consistent QT prolongation a better description might be that of an arrhythmogenic channelopathy (a point of possibly broader application). Be that as it may, clearly, *CACNA1C* families need to be advised on the basis of their own specific variant.

## CONFLICT OF INTEREST

The authors declare no conflict of interests.
