# Supplementary material for: Penetrance and expressivity of the R858H CACNA1C variant in a five‐generation pedigree segregating an arrhythmogenic channelopathy
Source: Mol Genet Genomic Med. 2018 Oct 21;7(1):e00476. doi: 10.1002/mgg3.476 (PMC6382452; doi:10.1002/mgg3.476)

Supplementary Online Material

• Representative EKG recordings (precordial leads V4-V6) of all family members in whom these archived data were available

• Shock electrograms (IV:32)

• Holter report (IV:39)

II:8 (QTc = 445 msec)


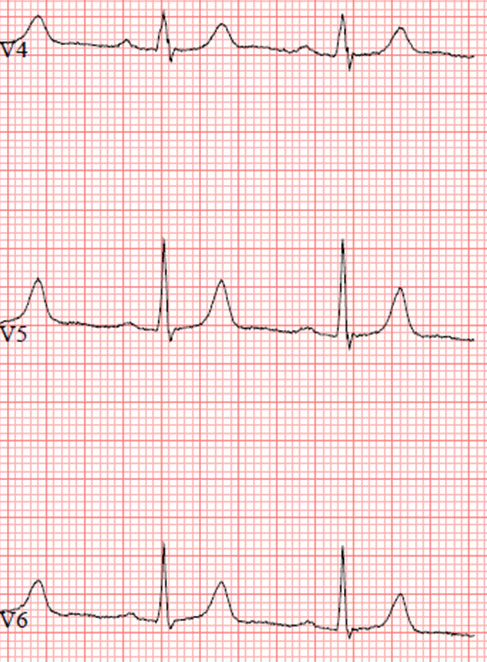


III:2 (QTc = 449 msec) Age 58 yr


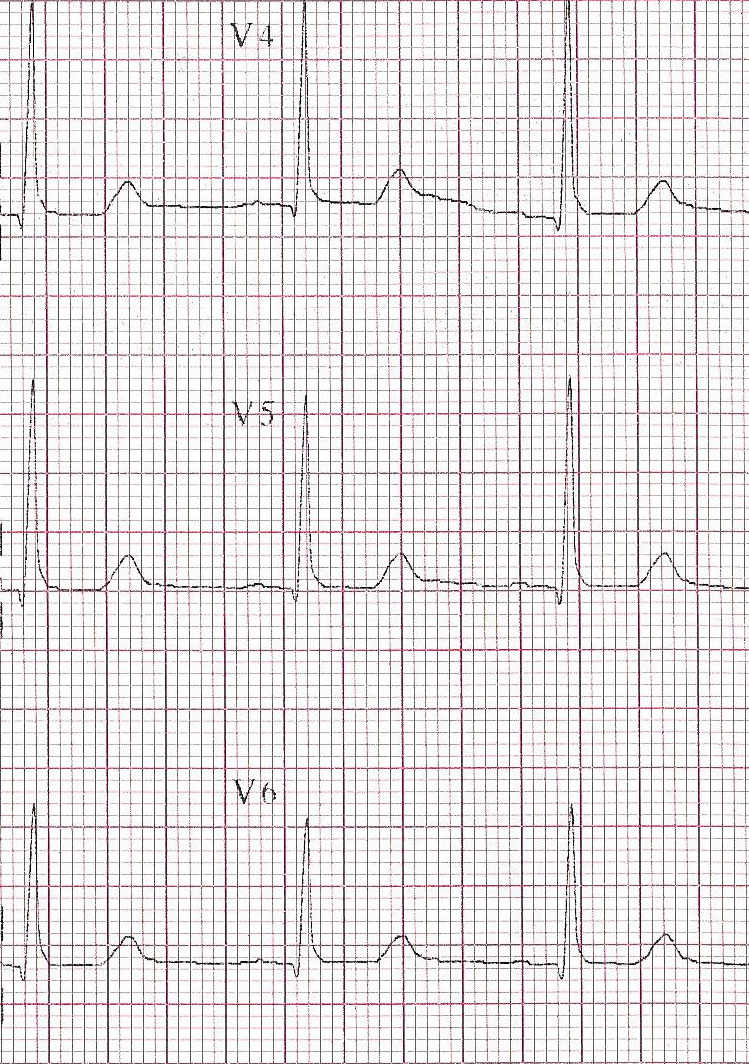


III:2 (QTc = 470 msec; left bundle branch block) Age 66 yr


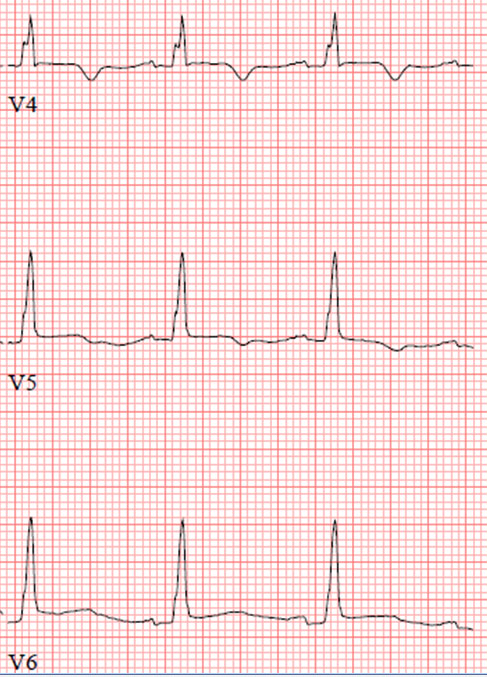


III:2 (QTc = 738 msec) On this occasion, at age 68 yr, a concomitant acute myocardial infarction


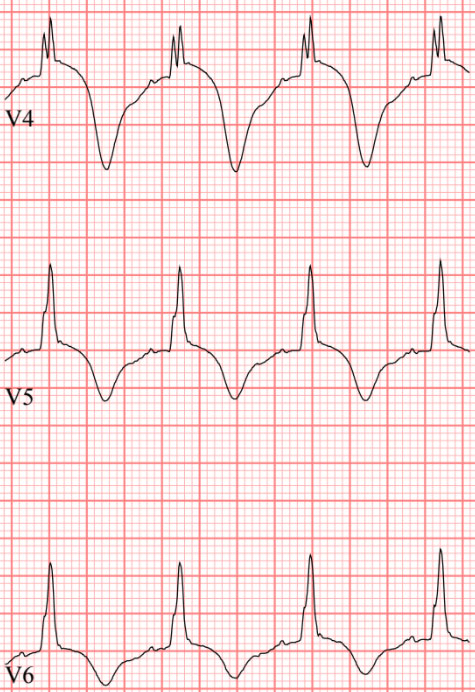


III:3 (QTc = 495 msec)


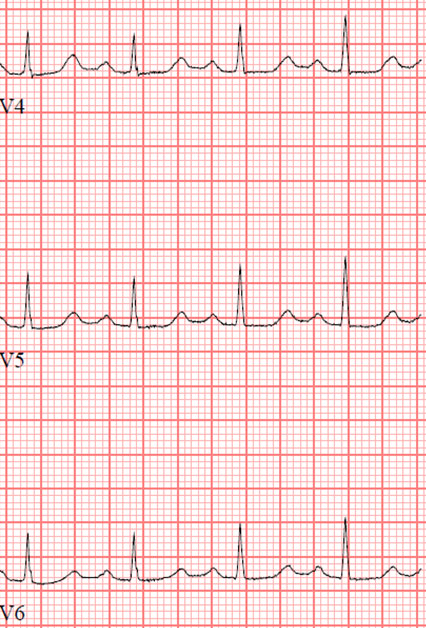


III:4 (QTc = 466 msec)


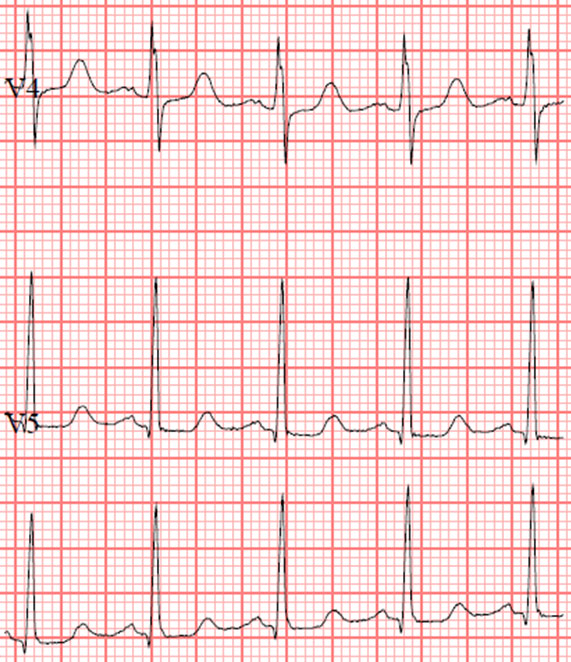


III:17 (QTc = 472 msec)


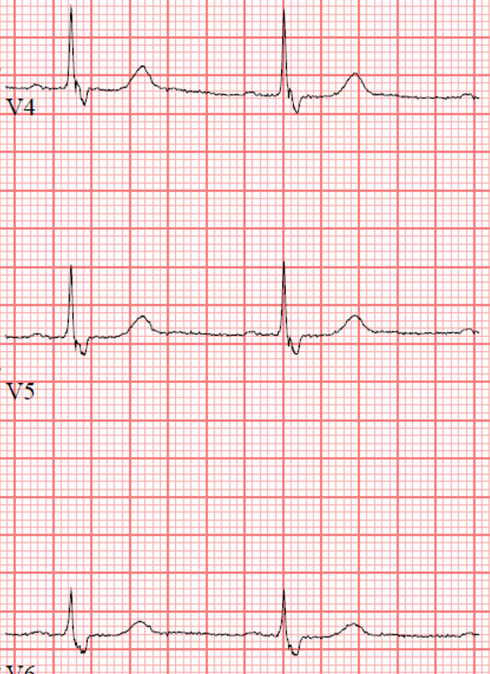


III:18 (QTc = 450 msec)


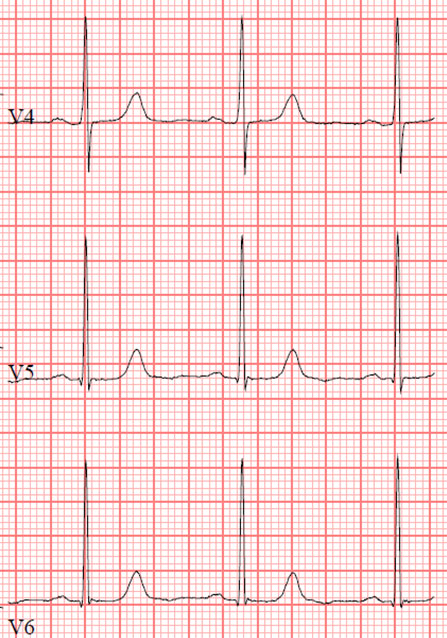


III:22 (QTc = 431 msec)


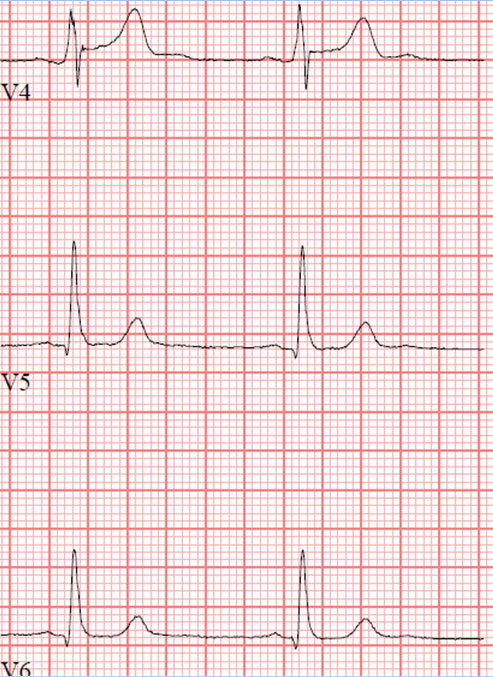


IV:6 (QTc = 480 msec)


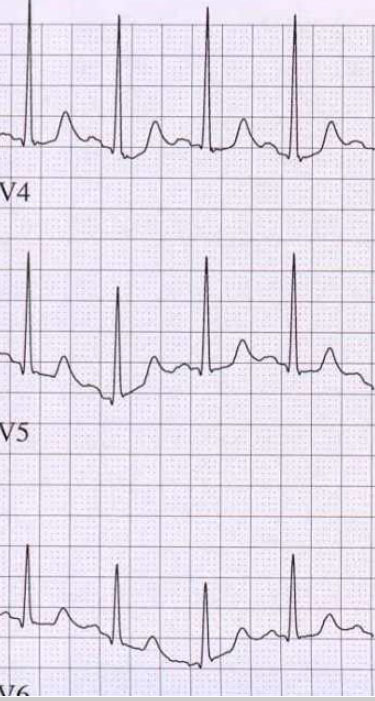


IV:11 (QTc = 473 msec)


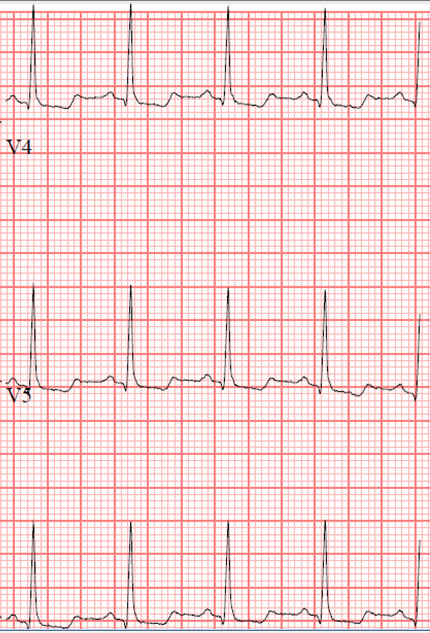


IV:30 (QTc = 454 msec)


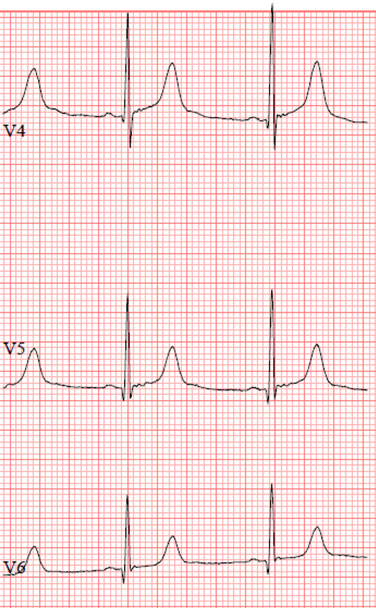


IV:31 (QTc = 429 msec)


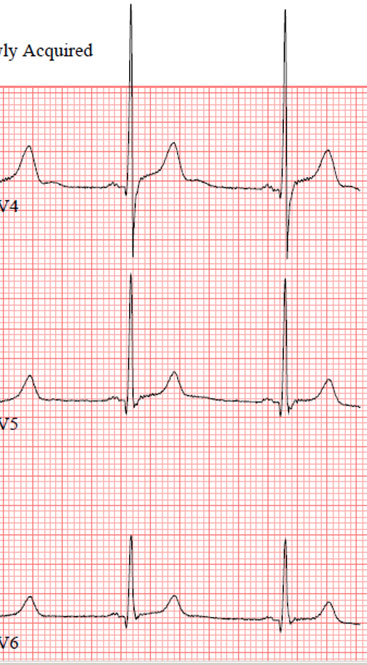


IV:32, index patient (QTc = 449 msec)


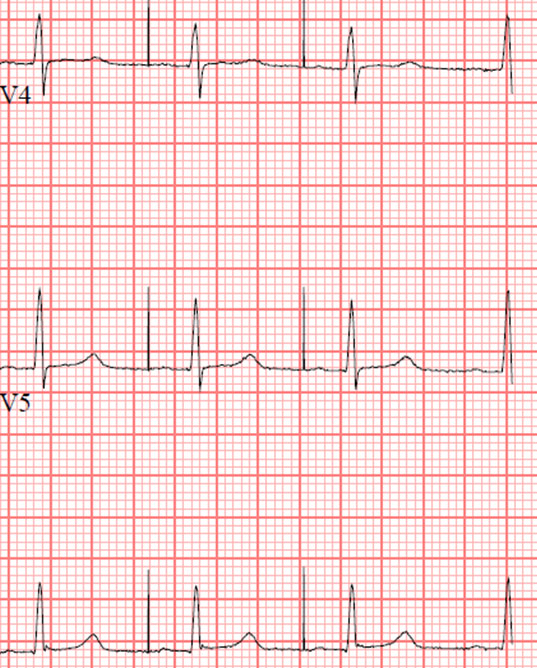


IV:32, index patient Shock delivered by ICD, first available recorded event from archived data


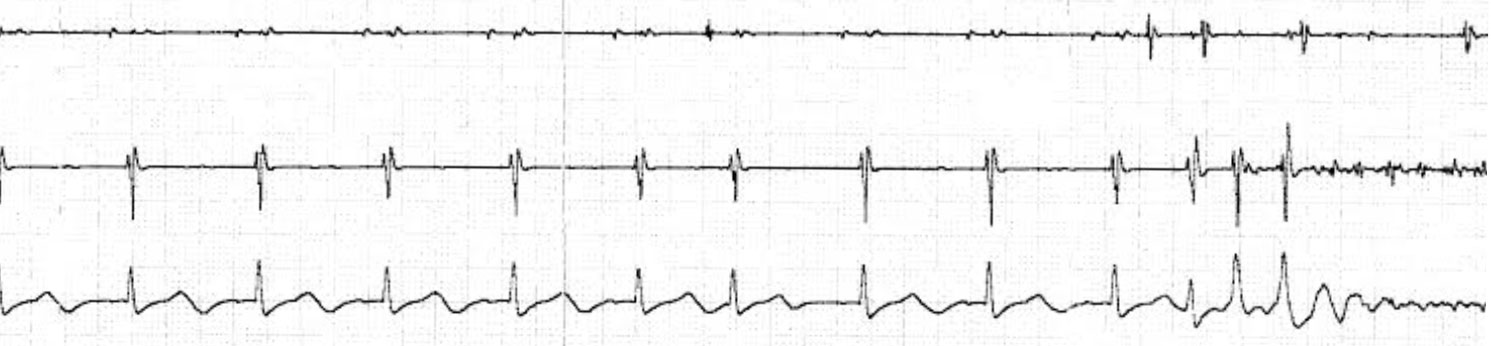


First row, atrial electrogram; second row, ventricular electrogram; third row, shock electrogram. Note considerable Q-T elongation in the shock electrogram (if the negative phase of the T wave is included), which was never otherwise seen on multiple routine surface EKGs.

IV:32, index patient Shock delivered by ICD, second available recorded event


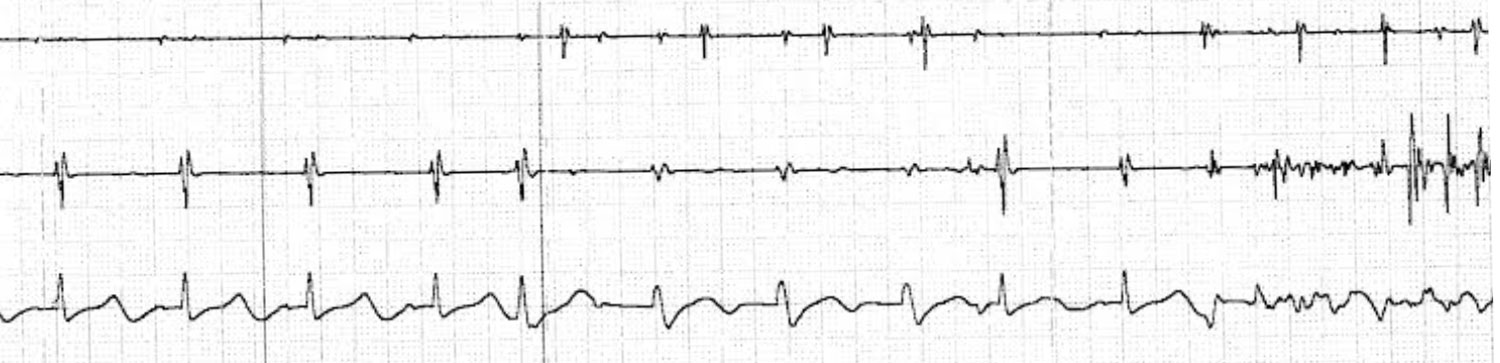


IV:33 (QTc = 502 msec)


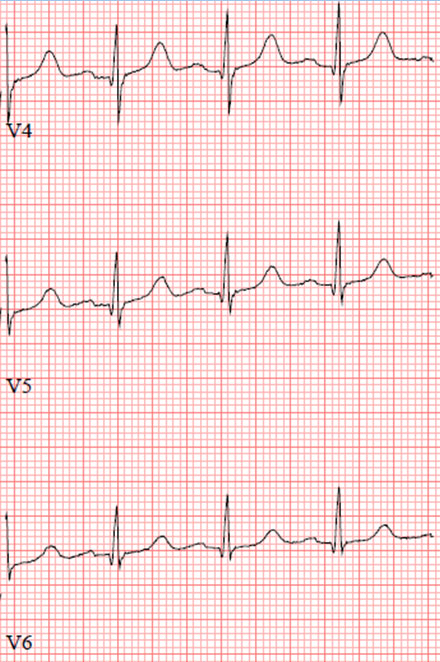


IV:34 (QTc = 476 msec)


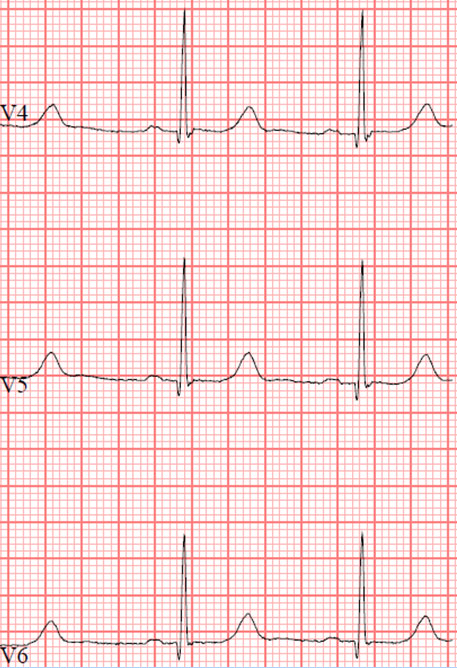


IV:39 Holter report at age 25 yr (no actual EKG tracing available for review)


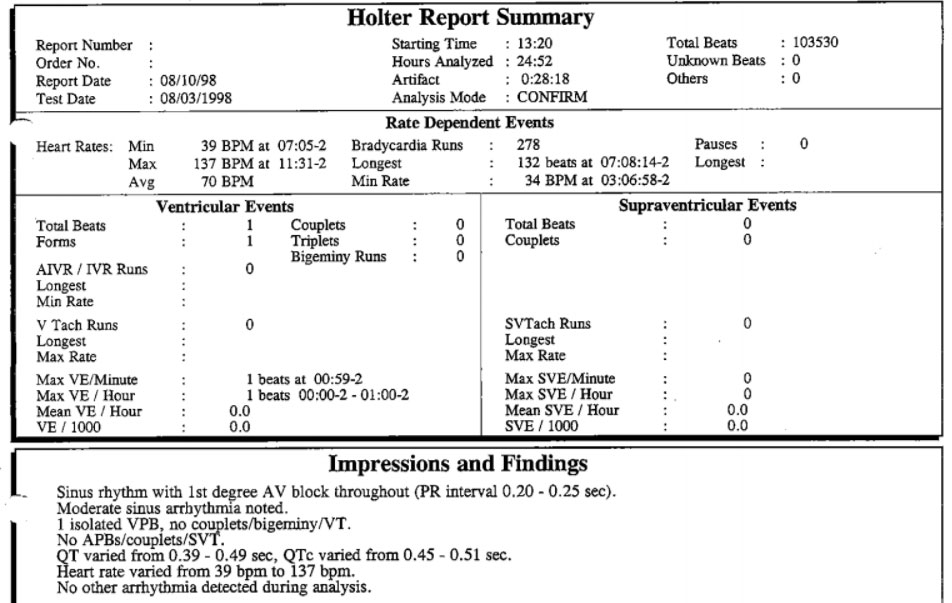


V:9 (QTc = 475 msec)


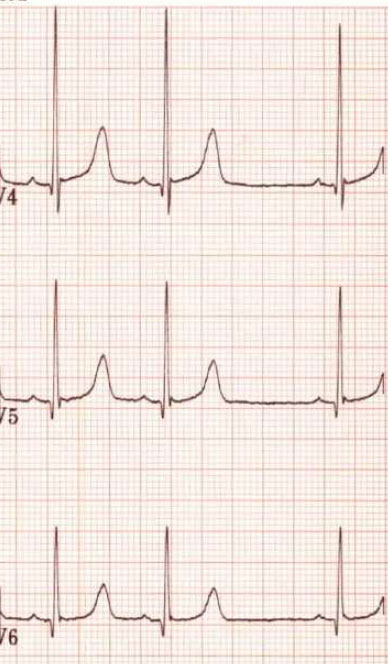


V:14 (QTc = 422 msec)


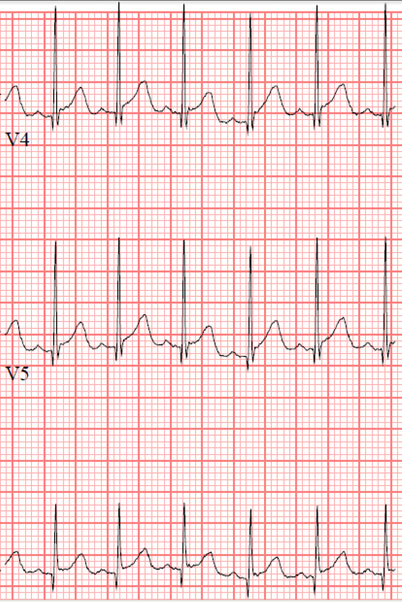


V:15 (QTc = 437 msec)


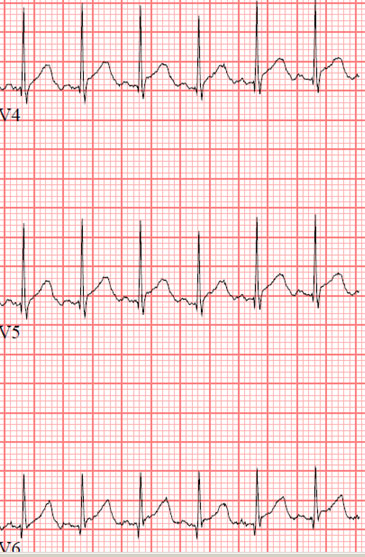


V:16 (QTc = 469 msec)


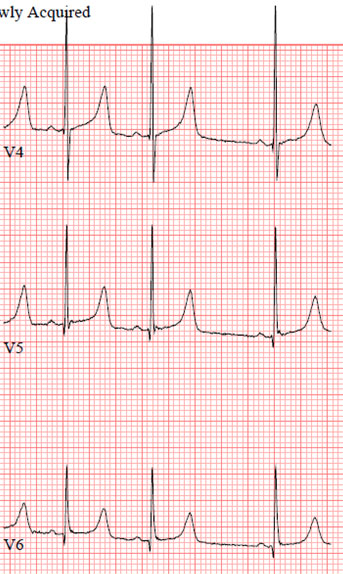


V:19 (QTc = 457 msec; tetralogy of Fallot)


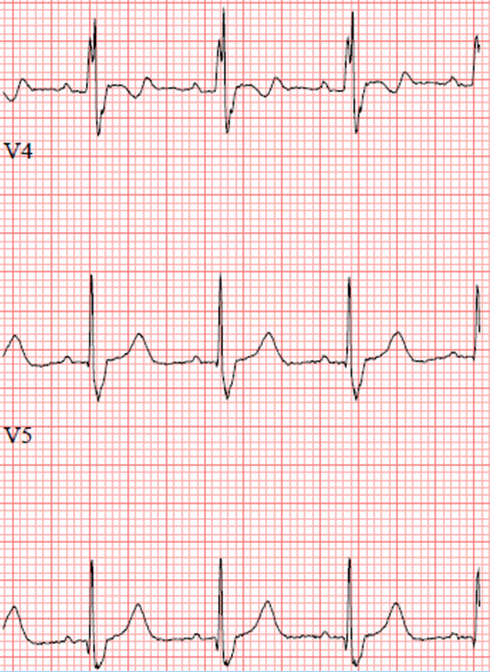


V:21 (QTc = 414 msec)


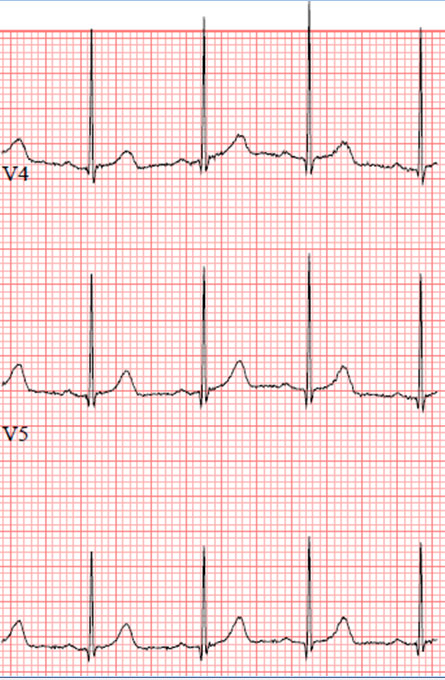


V:25 (QTc = 434 msec)


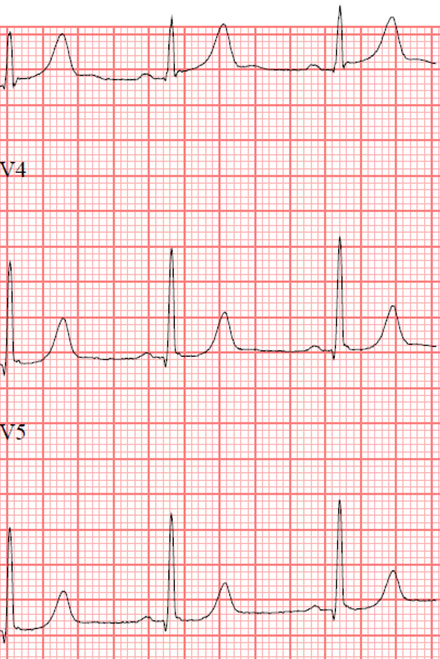

Supplement: Supplementary file 1 [file MGG3-7-na-s001.docx]
